# Obesity and pregnancy outcomes: Do the relationships differ by maternal region of birth? A retrospective cohort study

**DOI:** 10.1186/s12884-016-1087-5

**Published:** 2016-09-29

**Authors:** Miranda Davies-Tuck, Joanne C. Mockler, Lynne Stewart, Michelle Knight, Euan M. Wallace

**Affiliations:** 1 The Ritchie Centre, Hudson Institute of Medical Research, Clayton, Vic 3168 Australia; 2 Monash Health, Monash Medical Centre, Clayton, Australia; 3 Department of Obstetrics and Gynaecology, Monash University, Clayton, VIC Australia

**Keywords:** Obesity, Maternal region of birth and Pregnancy outcomes

## Abstract

**Background:**

We aimed to determine whether the association between obesity and a range of adverse maternal and perinatal outcomes differed in South Asian and Australian and New Zealand born women.

**Methods:**

A retrospective cohort study of singleton births in South Asian (SA) and Australian/New Zealand (AUS/NZ) born women at an Australian hospital between 2009 and 2013. The interaction between maternal region of birth and obesity on a range of maternal and perinatal outcomes was assessed using multivariate logistic regression.

**Results:**

Obesity was more strongly associated with gestational hypertension/Preeclampsia/HELLP and Gestational Diabetes Mellitus in AUS/NZ born women (*p* = 0.001 and *p* < 0.001, respectively for interaction) and was only associated with shoulder dystocia in SA born women (*p* = 0.006 for interaction). There was some evidence that obesity was more strongly related with admission to NICU/Special care nursery (SCN) (*p* = 0.06 for interaction) and any perinatal morbidity (*p* = 0.05 for interaction) in SA born women.

**Conclusions:**

Interventions targeted at reducing maternal obesity will have different impacts in SA compared to AUS/NZ born women.

## Background

Maternal obesity has emerged as one of the key contributors to adverse pregnancy outcomes in high-income nations [1], with no evidence that this trend is likely to reverse in the near future. In these countries almost half of women enter pregnancy with a body mass index (BMI) of 25 or more [1]. Interestingly, many of the adverse outcomes associated with maternal obesity, such as stillbirth, gestational diabetes mellitus (GDM), and operative delivery [1], are also more common in Asian women, with some of the highest rates of poor outcome seen in south Asian(SA) born women with obesity [2, 3]. Not surprisingly, it has been suggested that the associations between obesity and adverse pregnancy outcomes may be additive in some ethnicities [2–4]. For example, in a study of singleton births in London, UK, obesity in Asian (South and other Asian) women was associated with a stillbirth rate five times higher than in Asian women without obesity. The rate was lower still in Caucasian women, irrespective of obesity [2]. Similarly, obesity appears to be a stronger risk factor for GDM in Asian women than in Caucasian women [3]. However, none of these studies examined possible differential associations between obesity and other maternal and perinatal outcomes by maternal Asian ethnicity. Further, the patterns of Asian migration in the UK have been quite different to that elsewhere in the world and so whether the findings there are equally applicable to Australia is not known. This is potentially quite important because both migration from South Asia to Australia and other high-income countries outside the UK and, quite separately, the rate of obesity among SA born women are increasing internationally [5–8] (http://www.statcan.gc.ca/pub/89-621-x/89-621-x2007006-eng.htm). Accordingly, we undertook this study to determine whether the association between maternal obesity and a range of adverse maternal and perinatal outcomes differed in South Asian and Australian born women.

## Methods

We studied all singleton births ≥24 weeks gestation, free from congenital anomalies at Monash Women’s Services, Monash Health, a metropolitan maternity service in Melbourne, Australia, from 2009 to 2013, inclusive. Data were extracted from the Birthing Outcomes System, an electronic database recording all births ≥20 weeks’ gestation. For woman the attending midwife, supported by routine data validation, enters 191 data items over the course of pregnancy. For this study, we extracted data from the following fields: maternal age, self reported pre-gravid at booking body mass index (BMI), region of birth, parity, smoking, private or public care, obstetric and intrapartum complications (e.g fetal compromise), onset of labour (spontaneous or induced), augmentation of labour, epidural use, length of first and second stage of labour, blood loss, gestation at birth, birth weight, baby gender, mode of birth (spontaneous/instrumental/caesarean (further defined as Planned or Unplanned), admission to NICU/SCN, perinatal morbidity (e.g. respiratory distress, bradycardia, sepsis, meconium aspiration, birth trauma and birth asphyxia) and stillbirth. Fields were largely complete. Only women who had a pre-gravid BMI at booking recorded were included (98.5 % of AUS/NZ and 99.5 %% of SA women). Other missing data were case-wise excluded. The only fields with missing data were blood loss (0.1 %, *n* = 15) and baby gender (0.015 %, *n* = 4) all other fields were 100 % complete. The birthing outcome system only collects information on self-reported maternal region of birth. Therefore this was then classified, as either Australian and New Zealand (AUS/NZ) region of birth or South Asian (e.g. Afghanistan, Bangladesh, Bhutan, India, Iran, Maldives, Nepal, Pakistan and Sri Lanka) region of birth, according to the United Nations regional groups [9] and was used as a proxy marker for ethnicity.

Women of other nationalities were excluded because the aim of the study was to examine outcomes in South Asian women relative to AUS/NZ women. Obesity was defined as a BMI ≥ 30 kg/m^2^. The study was granted an exemption from ethics review by the Monash University Human Research and Ethics Committee, as per section 5.1.22 of the National statement on ethical conduct in Human Research 2007 [10].

### Statistical analysis

Maternal demographics, pregnancy, labour and baby outcomes were tabulated by maternal region of birth and obesity. Differences in demographics across groups were determined by a chi^2^ test. The univariate association between maternal obesity and region of birth group and pregnancy, labour and birth outcomes were assessed using logistic regression. Known and potential risk factors that were assessed for their inclusion in the final model were maternal age, parity, patient account class, smoking, onset of labour (spontaneous, induced, no labour), gestation, baby birth weight, baby gender, augmentation, epidural, placental abnormality, baby birth weight, onset of labour, birth type (e.g. vaginal/instrumental/operative), episiotomy, length of labour, pre-existing maternal medical conditions and previous caesarean. A number of potential confounders may also reflect steps in the causal pathway, e.g. Obesity to Pre-existing Diabetes to Gestational diabetes, therefore based on the literature potential intermediaries were not included in the final regression models. Potential co-linearity between confounders was also determined prior to the final model being defined. Each of the confounders included in the final model are detailed in the table footnotes. The interaction between maternal region of birth and obesity on each of the outcomes was then assessed by computing an interaction term and including it in the model. Logistic regression was then performed, stratified for maternal country of birth. The likelihood ratio was used to determine the final model. Differential BMI cut offs for SAs have been recommended by the World Health Organisation [11]. Therefore, the analysis was also undertaken defining obesity in South Asian women as ≥26 kg/m^2^. Doing so did not change our findings. Therefore, we present results using a BMI cut off of 30 kg/m^2^ only. Non-independence is a recognised issue within perinatal datasets, therefore all analyses were also run in nulliparous women only. This did not alter the associations. Due to the rare nature of some of the outcomes we therefore present data on all women to preserve power. Due to the number of hypotheses tested, we also computed a Benjamini–Hochberg false discovery rate corrected p value, after doing this a *p*-value <0.046 (two-tailed) was regarded as significant. All analyses were performed using the SPSS statistical package (SPSS 20, IBM Corp, Armonk, New York, USA).

## Results

Between 2009 and 2013 there were 41 041 singleton births at our institution. Of these, 18 768 (45 %) were to women born either in Australia or New Zealand (AUS/NZ) and 8342 (20 %) were to women born in South Asian countries (SA). Indian women comprised the majority of SA born women (51.4 %), followed by Sri Lankan (21.2 %) and Afghan women (18.6 %). Obesity was seen 27 % of AUS/NZ born women and 10 % of SA born women. The characteristics of the women, stratified by maternal region of birth and obesity, are summarised in Table 1. Associations between pregnancy, labour and perinatal outcomes and maternal region of birth and obesity are summarised in Table 2. Australian born mothers with obesity had the highest rates of gestational hypertension/PE/HELLP, PPH, induced labours and macrosomic babies. Women born in SA with obesity had the highest rates of Gestational Diabetes, Dystocia, unplanned caesarean, fetal compromise, admission to NICU/SCN and any perinatal morbidity. Overall compared to Australian born women without obesity, SA born women with obesity were 7.4 times (95 % CI 6.1–9.02) more likely to have gestational diabetes (*p* < 0.001), twice as likely (95 % CI 1.37–3.01) to experience dystocia (*p* < 0.001), twice as likely (95 % CI 1.66–2.29) to require an unplanned caesarean (*p* < 0.001), 39 % more likely (1.20–1.61) to experience fetal compromise, 53 % (95 % CI 1.31–1.79) more likely to have a baby admitted to the NICU and 39 % (95%CI 1.21–1.59) more likely to have a baby experience a perinatal morbidity (*p* < 0.001) The highest rates of stillbirth were also seen in women born in SA with obesity, although this difference was not statistically significant.

**Table 1:** Description of study population

	Australian mothers without obesity (*n* = 13,605)	Australian mothers with obesity (*n* = 5163)	South Asian mothers without obesity (*n* = 7467)	South Asian mothers with obesity (*n* = 875)
Maternal age groups				
< 20 years	621(4.6 %)	139(2.7 %)	34(0.5 %)	4(0.5 %)
20–30years	5846(43.0 %)	2289(44.3 %)	4062(54.4 %)	345(39.4 %)
> 30 years	7138(52.5 %)	2735(53 %)	3371(45.1 %)	526(60.1 %)
Nulliparous	6131(45.1 %)	1887(36.5 %)	3883(52 %)	307(35.1 %)
Past caesarean	1576(11.6 %)	1007(19.5 %)	942(12.6 %)	185(21.1 %)
Private patient	2616(16.2 %)	584(11.3 %)	587(7.9 %)	57(6.5 %)
Smoking				
Smoker	2259(16.6 %)	932(18.1 %)	24(0.3 %)	2(0.2 %)
Non-Smoker	9964(73.2 %)	3625(70.2 %)	7407(99.2 %)	860(98.3 %)
Spontaneous quitter	1382(10.2 %)	606(11.7 %)	36(0.5 %)	13(1.5 %)
Pre-existing hypertension	96(0.7 %)	208(4.0 %)	30(0.4 %)	15(1.7 %)
Pre-existing diabetes	162(1.2 %)	121(2.3 %)	60(0.8 %)	29(3.3 %)
Pre-existing thyroid disease	284(2.1 %)	146(2.8 %)	410(5.5 %)	68(7.8 %)
Baby gender-male	6952(51.1 %)	2676(51.8 %)	3857(51.7 %)	438(50.1 %)
Gestational age				
< 37 weeks	1256(9.2 %)	501(9.7 %)	441(5.9 %)	55(6.3 %)
37–41 + 6 weeks	12,115(89 %)	4570(88.5 %)	6927(92.8 %)	805(92 %)
≥ 42 weeks	234(1.7 %)	92(1.8 %)	99(1.3 %)	15(1.7 %)

Number(%)

Chi^2^ test to determine differences across the four groups. All differences were statistically significant at the *p* < 0.001 level

Obesity defined as BMI ≥30 kg/m^2^

**Table 2:** Pregnancy, labour and baby outcomes by maternal region of birth and obesity

	Number(%)	Crude odds ratio (95 % CI)	*P* value
Gestational Hypertension/PE/HELLP^1^ Syndrome			
Australian mothers without obesity	583(4.3 %)	1	-
Australian mothers with obesity	586(11.3 %)	2.86(2.54–3.22)	**<0.001**
South Asian mothers without obesity	264(3.5 %)	0.82(0.71–0.95)	**0.008**
South Asian mothers with obesity	45(5.1 %)	1.21(0.89 to 1.65)	0.23
Gestational Diabetes			
Australian mothers without obesity	433(3.2 %)	1	-
Australian mothers with obesity	491(9.5 %)	3.20(2.80–3.65)	**<0.001**
South Asian mothers without obesity	794(10.6 %)	3.62(3.21–4.08)	**<0.001**
South Asian mothers with obesity	172(19.7 %)	7.44(6.14–9.02)	**<0.001**
Preterm Birth			
Australian mothers without obesity	1256(9.2 %)	1	-
Australian mothers with obesity	501(9.7 %)	1.06(0.95–1.18)	0.32
South Asian mothers without obesity	441(5.9 %)	0.62(0.55–0.69)	**<0.001**
South Asian mothers with obesity	55(6.3 %)	0.66(0.50–0.87)	**0.003**
Dystocia			
Australian mothers without obesity	226(1.7 %)	1	-
Australian mothers with obesity	101(2.0 %)	1.18(0.93–1.50)	0.17
South Asian mothers without obesity	117(1.6 %)	0.94(0.75–1.18)	0.61
South Asian mothers with obesity	29(3.3 %)	2.03(1.37–3.01)	**<0.001**
Postpartum Haemorrhage 1000 ml			
Australian mothers without obesity	661(4.9 %)	1	-
Australian mothers with obesity	372(7.2 %)	1.52(1.33–1.73)	**<0.001**
South Asian mothers without obesity	334(4.5 %)	0.92(0.80–1.05)	0.20
South Asian mothers with obesity	49(5.6 %)	1.16(0.86–1.56)	0.33
Induced Labour			
Australian mothers without obesity	2955(21.7 %)	1	-
Australian mothers with obesity	1496(29 %)	1.47(1.37–1.58)	**<0.001**
South Asian mothers without obesity	1727(23.1 %)	1.09(1.01–1.16)	**0.02**
South Asian mothers with obesity	244(27.9 %)	1.39(1.20–1.62)	**<0.001**
Instrumental Vaginal			
Australian mothers without obesity	1846(13.6 %)	1	-
Australian mothers with obesity	478(9.3 %)	0.65(0.58–0.72)	**<0.001**
South Asian mothers without obesity	1284(17.2 %)	1.32(1.24–1.43)	**<0.001**
South Asian mothers with obesity	97(11.1 %)	0.79(0.64–0.99)	**0.04**
Unplanned Caesarean			
Australian mothers without obesity	1898(14 %)	1	-
Australian mothers with obesity	1003(19.4 %)	1.49(1.37–1.62)	**<0.001**
South Asian mothers without obesity	1398(18.7 %)	1.42(1.32–1.53)	**<0.001**
South Asian mothers with obesity	210(24 %)	1.95(1.66–2.29)	**<0.001**
Small for gestational age(<10th centile)			
Australian mothers without obesity	1449(10.7 %)	1	-
Australian mothers with obesity	367(7.1 %)	0.64(0.57–0.72)	**<0.001**
South Asian mothers without obesity	1262(16.9 %)	1.71(1.57–1.85)	**<0.001**
South Asian mothers with obesity	90(10.3 %)	0.96(0.77–1.20)	0.73
Macroscomia(>4 kg)			
Australian mothers without obesity	1575(11.6 %)	1	-
Australian mothers with obesity	994(19.3 %)	1.82(1.67–1.99)	**<0.001**
South Asian mothers without obesity	439(5.9 %)	0.48(0.43–0.53)	**<0.001**
South Asian mothers with obesity	111(12.7 %)	1.11(0.90–1.36)	0.32
Fetal compromise (pregnancy or labour)			
Australian mothers without obesity	3431(25.2 %)	1	-
Australian mothers with obesity	1372(26.6 %)	1.07(1.00–1.15)	0.06
South Asian mothers without obesity	2315(31 %)	1.33(1.25–1.42)	**<0.001**
South Asian mothers with obesity	279(31.9 %)	1.39(1.20–1.61)	**<0.001**
Admission to NICU/SCN^2^			
Australian mothers without obesity	2664(19.5 %)	1	-
Australian mothers with obesity	1238(24.1 %)	1.32(1.22–1.42)	**<0.001**
South Asian mothers without obesity	1455(19.6 %)	1.01(0.94–1.08)	0.85
South Asian mothers with obesity	235(27 %)	1.53(1.31–1.79)	**<0.001**
Any Perinatal Morbidity			
Australian mothers without obesity	5726(42 %)	1	-
Australian mothers with obesity	2341(45.3 %)	1.14(1.07–1.22)	**<0.001**
South Asian mothers without obesity	3197(42.8 %)	1.03(0.97–1.09)	0.31
South Asian mothers with obesity	49(50.2 %)	1.39(1.21–1.59)	**<0.001**
Stillbirth			
Australian mothers without obesity	72(0.5 %)	1	-
Australian mothers with obesity	31(0.6 %)	1.14(0.74–1.73)	0.56
South Asian mothers without obesity	32(0.4 %)	0.81(0.55–1.23)	0.32
South Asian mothers with obesity	6(0.7 %)	1.30(0.56–2.99)	0.54

Number in bold reflect statistical significance

Number(%)

^1^Pre-eclampsia(PE)

^2^Neonatal intensive care unit(NICU)/ Special care nursery(SCN)

The associations between obesity and pregnancy and labour outcomes, stratified for maternal region of birth, are presented in Table 3. The association between obesity and hypertension/PE/HELLP and GDM was stronger in AUS/NZ born women than in SA born women (*p* = 0.001 and *p* < 0.001, respectively for interaction). Obesity was associated with a three-fold increased likelihood (95 % CI 2.78–3.54, *p* < 0.001) of gestational hypertension/PE/HELLP in AUS/NZ born women compared to only a 59 % increased likelihood (95 % CI 1.14–2.21, *p* = 0.006) in SA born women. Similarly, GDM was three times more common (OR 3.2, 95 % CI 2.80–3.67, *p* < 0.001) in AUS/NZ born women with obesity and nearly twice as common (OR 1.89(1.57–2.28), *p* < 0.001) in SA born women with obesity. Obesity was also associated with shoulder dystocia in SA born women but not in AUS/NZ born women (*p* = 0.006 for interaction). For all women, irrespective of maternal region of birth, obesity was associated with an increased odds of unplanned caesarean section. There was no evidence of effect modification by maternal region of birth for the association between obesity and preterm birth, severe post-partum haemorrhage, instrumental vaginal birth or unplanned caesarean section.

**Table 3:** Adjusted odds ratio (95 % CI) for pregnancy and labour outcomes according to maternal obesity

	Australian women odds ratio (95 % CI)	*P* value	South Asian Women odds ratio (95 % CI)	*P* value	P for interaction
Gestational Hypertension/PE/HELLP ^a^	3.14(2.78–3.54)	**<0.001**	1.59(1.14–2.21)	**0.006**	**0.001**
Gestational Diabetes ^b^	3.21(2.80–3.67)	**<0.001**	1.89(1.57–2.28)	**<0.001**	**<0.001**
Preterm Birth ^c^	1.04(0.93–1.16)	0.49	1.08(0.81–1.45)	0.60	0.99
Dystocia ^d^	1.11(0.87–1.41)	0.16	1.99(1.26–2.96)	**0.002**	**0.006**
PPH1000ml ^e^	1.49(1.30–1.71)	**<0.001**	1.32(0.96–1.82)	0.09	0.36
Instrumental Vaginal ^f^	0.73(0.65–0.82)	**<0.001**	0.76(0.57–1.01)	*0.05*	0.73
Unplanned Caesarean ^g^	1.35(1.23–1.49)	**<0.001**	1.38(1.15–1.66)	**0.001**	0.48

Number in bold reflect statistical significance

^a^ Odds ratio for Gestational Hypertension/PE/HELLP according to maternal obesity adjusted for age, parity and smoking status

^b^ Odds ratio for gestational diabetes according to maternal obesity adjusted for age, parity and smoking status

^c^ Odds ratio for preterm birth according to maternal obesity adjusted for age, parity and smoking

^d^ Odds ratio for shoulder dystocia according to maternal obesity adjusted for age, parity, induction, augmentation and epidural

^e^ Odds ratio for PPH1000mls according to maternal obesity adjusted age, parity, placental abnormality, baby birth weight, gestational hypertension/PE/HELLP, onset of labour, birth type (e.g. vaginal/instrumental/operative), episiotomy, Length of labour and pre-existing maternal blood disorder

^f^ Odds ratio for instrumental delivery according to maternal obesity adjusted for maternal age, parity, onset of birth, epidural, baby birth weight, gestation, head position, augmentation and account class

^g^ Odds ratio for Unplanned caesarean delivery according to maternal obesity adjusted for maternal age, parity, account class, previous ceasarean, onset of labour, gestation, birth weight, augmentation, epidural

The associations between obesity and infant outcomes, stratified for maternal region of birth, are presented in Table 4. There was some evidence that obesity was more strongly related with admission to NICU/SCN (*p* = 0.06 for interaction) and any perinatal morbidity (*p* = 0.05 for interaction) in SA born women. Obesity was associated with a 66 % increased likelihood of NICU/SCN admission (95 % CI 1.66(1.40–1.97)) in SA born women compared to a 33 % (95 % CI (1.22–1.45)) increase in AUS/NZ born women. Obesity was also associated with a 45 % (95 % CI 1.25–1.68)) increased likelihood of any perinatal morbidity in SA born women compared to a 18 % (95 % CI 1.04–1.27) increased likelihood of any perinatal morbidity in AUS/NZ born women. For all women, irrespective of maternal region of birth, obesity was associated with a reduced likelihood of a Small for Gestational Age (SGA; <10th centile) and an increased likelihood of macrosomia (4.5 kg or more) and fetal distress (Table 4). These findings remained the same when the lower BMI cut off for SA women was used (data not shown). There was no evidence of effect modification by maternal region of birth for obesity and small for gestational age, macrosomia, fetal distress in labour and stillbirth.

**Table 4:** Adjusted odds ratio (95 % CI) for baby outcomes according to obesity

	Australian women odds ratio (95 % CI)	*P* value	South Asian Women odds ratio (95 % CI)	*P* value	P for interaction
Small for gestational age ^a^	0.64(0.57–0.72)	**<0.001**	0.64(0.51–0.81)	**<0.001**	0.72
Macroscomia (>4 kg) ^b^	1.90(1.73–2.08)	**<0.001**	2.24(1.75–2.83)	**<0.001**	0.10
Fetal Distress (pregnancy or labour) ^c^	1.19(1.02–1.28)	**<0.001**	1.31(1.12–1.53)	**0.001**	0.38
Admission to NICU/SCN ^d^	1.33 (1.22–1.45)	**<0.001**	1.66(1.40–1.97)	**<0.001**	*0.06*
Any Perinatal Morbidity ^e^	1.18(1.04–1.27)	**<0.001**	1.45(1.25–1.68)	**<0.001**	*0.052*
Stillbirth ^f^	0.90(0.57–1.42)	0.65	1.42(0.55–3.63)	0.47	0.15

Number in bold reflect statistical significance

^a^ Odds ratio for small for gestation age baby according to maternal obesity adjusted for maternal age, parity, smoking and account class

^b^ Odds ratio for macrosomia according to maternal obesity adjusted for parity, maternal age, account class, smoking, gestation and baby gender

^c^ Odds ratio for Fetal Distress (pregnancy or labour) according to maternal obesity adjusted for maternal age, parity, smoking, gestation and baby gender

^d^ Odds ratio for baby admission to NICU/SCN according to maternal obesity adjusted for parity, account class, GDM, gestation, baby gender, onset of labour, birth type

^e^ Odds ratio for any perinatal morbidity according to maternal obesity adjusted for maternal age, parity, account class, gestation, baby gender, onset of labour, birth type

^f^ Odds ratio for stillbirth according to maternal obesity adjusted for maternal age, parity, previous caesarean, account class, baby gender, gestation and smoking

## Discussion

In this study we have explored the relative impacts of maternal region of birth, as a surrogate for ethnicity, on the association between obesity and rates of maternal and perinatal outcomes. We have shown that important outcomes significantly differed by both maternal region of birth and by obesity. Further, maternal region of birth influenced the association between obesity and hypertensive conditions of pregnancy, GDM, dystocia, admission of baby to NICU/SCN and perinatal morbidity. We believe that these observations can be useful to clinicians, allowing better identification of ‘at risk’ pregnancies and individualisation of care in pregnancy and childbirth.

To our knowledge, this is the first study to examine the potential effect modification of maternal south Asian region of birth on the well established associations between maternal obesity and a range of maternal and perinatal outcomes in Australia. As such, our study is the first to explore whether the observations of similar studies previously undertaken in the United Kingdom [2–4] are evident in another international population. This is worth exploring because the ethnic composition of populations of SA women studied in the UK are different to those in Australia, the United States and Canada [6–8] (http://www.statcan.gc.ca/pub/89-621-x/89-621-x2007006-eng.htm). Specifically, the majority of SA born women in the previous UK study were from Pakistan and Bangladesh, with a minority from India [12]. In our study, Indian women comprised the majority of SA born women, followed by Sri Lankan and Afghan women. This is similar to the composition of SA born women in the USA and Canada [8] (http://www.statcan.gc.ca/pub/89-621-x/89-621-x2007006-eng.htm). This is potentially important because the altered rates of adverse pregnancy outcomes in SA born women have been reported to differ by country within SA [12].

Consistent with previous studies [3, 4] we found that rates of GDM were highest in SA born women with obesity compared to all other women, highlighting the value of appropriate weight management in pregnancy and early GDM testing [13, 14] in these women. However, the association between obesity and GDM was actually stronger in AUS/NZ born women, a finding that does not accord with previous reports from the UK [3, 4]. In those studies the relationship between obesity and diabetes was stronger in Asian and South East/East Asian (oriental) women than in Caucasian women [3, 4]. It is not clear why our findings differ but may reflect biological differences given the compositional make of South Asian born women in our study are different to those in the UK studies, future studies uncovering the mechanisms are needed. Nonetheless, they highlight that maternal obesity is an important risk factor for GDM in all women, irrespective of ethnicity.

The association between obesity and hypertensive disorders of pregnancy was also stronger in AUS/NZ born women than in SA born women, in whom the overall rate was lower. While the precise mechanisms by which obesity increases a woman’s risk of hypertension in pregnancy are poorly understood, the association between obesity and pregnancy hypertension has been observed worldwide, in developing and developed countries alike ([1, 15]. Indian women experience decreased rates of hypertension in pregnancy, regardless of obesity [16] suggesting that our finding may reflect a reduced susceptibility in South Asian born women to hypertensive conditions. Preeclampsia remains a major cause of maternal morbidity and mortality and iatrogenic preterm birth. This consistent observation emphasises the maternal and child health benefit opportunities that could be afforded by targeting pre-pregnancy weight and gestational weight gain management. In that regard, our findings suggest that interventions aimed at reducing maternal obesity will have a larger impact in AUS/NZ born women then in SA born women.

While not statistically significantly different, the rate of stillbirth was highest in the SA born women with obesity. Both obesity [1] and SA ethnicity [16–19] are recognised risk factors for stillbirth. This current study suggests that they may interact, an observation previously made by some [4] but not other investigators [2]. Our study was too small, and so underpowered, to be definitive.

The association between maternal obesity and shoulder dystocia is contentious [20]. We found that obesity was only associated with shoulder dystocia in SA born women. To our knowledge this has not been reported before. We identified that obesity was associated with macrosomia in all women regardless of maternal ethnicity suggesting this may not be the sole driver. Pelvimetry studies have demonstrated that South Asian born women have a smaller pelvic inclination than other women [21]. It has also been suggested that obesity leads to an increase in maternal soft tissue inside the pelvis, which narrows the birth canal [22]. These factors combined may explain why obesity was associated with shoulder dystocia in SA born women only. However future work is needed.

We also showed that obesity was more strongly related with admission to NICU/SCN and any perinatal morbidity in SA born women than in AUS/NZ born women. Why this was the case is not clear. The most frequent neonatal morbidities in our study were suspected sepsis, meconium aspiration and birth trauma. It has been previously shown that maternal obesity increases the risk of neonatal sepsis, patent ductus arteriosus, and/or necrotising enterocolitis [23], possibly through mechanisms of increased systemic inflammation [24, 25]. However, whether maternal ethnicity compounds those risks has not been previously reported.

Regardless of maternal region of birth, we also identified that obesity was associated with suspected fetal compromise, unplanned cesarean section and post partum hemorrhage (PPH) and was protective of instrumental vaginal birth. These findings are not surprising. Obesity has been consistently associated with cesarean section, with fetal compromise being a major driver for that increased risk [1, 26]. That obesity was protective for instrumental vaginal birth in the current study likely reflects the increased rate of caesarean section, resulting in fewer vaginal births overall.

Our study has a number of limitations. Due to how perinatal data are recorded in Australia we are only able to define ethnicity by maternal region of birth. It is possible that some women within the AUS/NZ born group are of south Asian ethnicity. However, this is likely to have underestimated the associations rather than overestimated them. Further, while obesity is an important risk factor, gestational weight gain is increasingly being recognised as an independent risk factor for perinatal outcomes [27–29]. Weight gain is not currently recorded in our electronic database and so we were unable to assess this. The exposures of interest in our study, maternal region of birth and obesity both exist prior to the outcomes occurring however it is possible that our findings only reflect an association not causation. It is also possible that unknown or unmeasured confounding could explain our findings. For example, vitamin D deficiency was not available for women in this study. Vitamin D deficiency is associated with both south Asian region of birth and obesity and has been suggested to be associated with dystocia, although the findings are not consistent [30]. Caution should be made with interpreting this finding.

## Conclusion

In summary, we have shown that maternal south Asian region of birth influences the established and well known associations between maternal obesity and hypertensive disorders of pregnancy, GDM, shoulder dystocia, admission of baby to NICU/SCN and perinatal morbidity. Accordingly, interventions targeted at reducing maternal obesity would be expected to have different impacts in SA born compared to AUS/NZ born women. Future research is needed to elucidate the specific mechanisms by which obesity is having differential effects and to assess the efficacy of such interventions on reducing adverse outcomes in women of differing ethnicities. Health economic modelling of cost: benefit analyses assessing interventions aimed at reducing obesity would need to take this into account.

## Abbreviations

AUS/NZ: Australian/New Zealand
BMI: Body mass index
GDM: Gestational Diabetes Mellitus
NICU: Neonatal Intensive Care Unit
PE: Preeclampsia
PPH: Post-partum hemorrhage
SA: South Asian
SGA: Small for gestational age
